# Pediatric Systemic Lupus Erythematosus Complicated by Acute EBV and CMV Co‐infection

**DOI:** 10.1002/jcla.70330

**Published:** 2026-08-14

**Authors:** Anning Chen, Gengchun Ji, Ziping Lan, Ping Li, Jianwei Wang, Le Zhang, Chaojun Hu, Shulan Zhang

**Affiliations:** ^1^ Department of Rheumatology Peking Union Medical College Hospital, Peking Union Medical College & Chinese Academy of Medical Sciences; National Clinical Research Center for Immunologic Diseases; Key Laboratory of Rheumatology and Clinical Immunology, Ministry of Education Beijing China; ^2^ Department of Laboratory Medicine, the Eighth Affiliated Hospital Sun Yat‐Sen University Shenzhen China; ^3^ Department of Clinical Laboratory, Shenzhen Baoan Hospital The Second Affiliated Hospital of Shenzhen University Shenzhen China; ^4^ Shenzhen Maternity and Child Healthcare Hospital, Women and Children's Medical Center Southern Medical University Shenzhen Guangdong Province China; ^5^ Department of Clinical Laboratory Fuzhou University Affiliated Provincial Hospital Fuzhou China; ^6^ Department of Medical Laboratory The Affiliated Huaian No. 1 People's Hospital of Nanjing Medical University Huaian Jiangsu China

**Keywords:** cytomegalovirus, Epstein–Barr virus, literature review, systemic erythematosus lupus

## Abstract

**Background:**

Systemic lupus erythematosus (SLE) is a multifactorial autoimmune disease characterized by multi‐organ damage, with genetic, environmental, and viral factors implicated in its pathogenesis. Herpesviruses such as Epstein–Barr virus (EBV) and cytomegalovirus (CMV) are known to trigger or exacerbate SLE, potentially through mechanisms like molecular mimicry and immune dysregulation.

**Case Presentation:**

We report the case of a 10‐year‐old girl who presented with a malar rash following sun exposure, accompanied by fever and arthritis. Laboratory investigations revealed the presence of multiple autoantibodies and serological evidence of active co‐infection with both EBV and CMV. A diagnosis of SLE with mild activity was established. The patient received intravenous methylprednisolone, belimumab, and oral ganciclovir. This led to significant clinical improvement, including resolution of the facial rash and normalization of joint mobility. Follow‐up assessments showed near‐complete normalization of inflammatory markers and autoantibody titers. However, causality cannot be established from a single case, and proposed mechanisms remain hypotheses.

**Conclusion:**

This case suggests that acute co‐infection with EBV and CMV may play a role in the pathogenesis of SLE, possibly via molecular mimicry and B‐cell activation. Therefore, routine screening for EBV and CMV in adolescent SLE patients may be warranted to advance etiological research and inform personalized treatment strategies.

## Graphical abstract

This case report describes a 10‐year‐old girl with new‐onset pediatric SLE who presented with malar rash, fever, and arthritis following sun exposure, alongside serological evidence of acute Epstein–Barr virus (EBV) and cytomegalovirus (CMV) co‐infection. She received intravenous methylprednisolone, belimumab, and oral ganciclovir, leading to significant clinical improvement with normalization of inflammatory markers and joint mobility. This case highlights the potential role of acute herpesvirus co‐infection in triggering SLE onset via immune dysregulation. It suggests that routine EBV/CMV screening in adolescent SLE patients may be valuable for guiding personalized immunosuppressive and antiviral therapy.

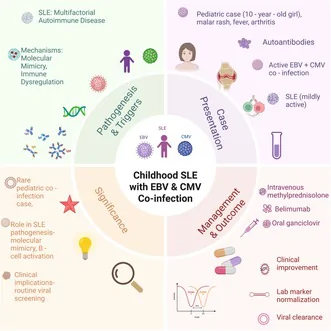

## 1 Introduction

Viral infections, particularly from the herpesvirus family, are recognized as significant environmental triggers for the onset and flares of systemic lupus erythematosus (SLE). Among these, Epstein–Barr virus (EBV) demonstrates one of the strongest associations with SLE [1]. EBV infection in children is predominantly observed during the preschool period (ages 3–6), with an infection rate exceeding 50%. Among young patients with SLE, a higher proportion have a history of prior EBV infection, suggesting that early viral exposure may trigger immune dysregulation and contribute to the development of SLE [2, 3]. The core pathogenic mechanisms may involve two key aspects: First, molecular mimicry. Specific sequences in EBNA‐1(e.g., PPRGRRP) exhibit homology with SLE autoantigens (Sm B/B′, Ro/SSA 60kD), inducing cross‐reactive antibodies that target host dsDNA [4]. Second, disruption of immune homeostasis. EBV drives aberrant B‐lymphocyte activation, while impaired control of latent infection in patients promotes viral reactivation and T‐cell depletion. Consequently, EBV proteins present in target organs (e.g., kidney tissue) directly mediate tissue injury [5]. Concurrently, acute CMV reactivation can induce SLE‐associated vascular lesions, exacerbate nephritis, and trigger macrophage activation syndrome (MAS).

This occurs via activation of the Toll‐like receptor (TLR) pathway, leading to the release of pro‐inflammatory cytokines (e.g., IFN‐α, TNF‐α). EBV and CMV thus form a synergistic “vicious cycle”, jointly inducing MAS and multi‐organ failure6. Based on these mechanisms, this study reports a case of SLE complicated by acute EBV and CMV co‐infections and provides a literature review.

## 2 Case Presentation

### 2.1 Clinical History

A 10‐year‐old girl was admitted to our hospital on April 8, 2025. Her illness began 20 days prior to admission following sun exposure, with the onset of an erythematous rash on both cheeks. Approximately 10 days later, the rash evolved into a classic malar (“butterfly”) erythema spanning the bridge of her nose. This was accompanied by the new onset of fever (maximum temperature 38.0°C) and arthritis, characterized by swelling and erythema of the proximal interphalangeal joints of her left hand. The patient denied any oral ulcers or alopecia. Her past medical and family histories were unremarkable. On physical examination, she had a distinct malar rash, scattered petechiae on her back, and an erythematous rash over the extensor aspect of the fourth proximal interphalangeal joint of her left hand. Musculoskeletal examination was notable for a positive Patrick's (FABER) test bilaterally.

### 2.2 Laboratory Findings

Initial laboratory results from the hospitalization (April 8–11, 2025) are summarized in Table 1. Key findings included significantly elevated levels and serological evidence of active infections with both EBV and CMV. Due to the unavailability of quantitative EBV DNA testing at admission, the diagnosis of acute EBV infection relied on serological criteria (positive VCA IgM and VCA IgA) combined with clinical features. For CMV, CMV PP65 antigenemia (9 positive cells/2 × 10^5^ leukocytes) was used as a semiquantitative marker of active infection. Serial follow‐up showed resolution of EBV IgM and reduction of CMV PP65 to 4 positive cells, paralleling clinical improvement. Immunophenotyping revealed a B‐lymphocyte proportion of 15.2% and a CD4+/CD8+ T‐cell ratio of 1.78. HLA‐B27 positive. There were no clinical features of spondyloarthritis, and this finding was considered incidental. Kidney function was normal and urinalysis showed no proteinuria or hematuria.

**TABLE 1:** Laboratory findings at admission and follow‐up.

Parameter	Unit	Reference range	At admission (Apr 2025)	At follow‐up (May 2025)
ANA (titer)	titer	< 1:80	1:1280 (Homogeneous + Cytoplasmic)	1:320 (Homogeneous + Speckled)
Anti‐dsDNA (IIF)	titer	< 1:10	1:20	1:10
Anti‐dsDNA (CLIA)	IU/mL	< 24.0	2684.3	119
ESR	mm/h	0–20	44	6
Anti‐RNP	AI	< 10	12.3	8.2
Anti‐rRNP	AI	< 10	27.2	5.6
Anti‐histone	AI	< 10	96	9
Anti‐nucleosome	AI	< 10	122	5.6
ACL IgG	GPLU/mL	< 8.0	12.4	3.8
Anti‐RA33	AU/mL	< 16.0	91.6	7.5
LF‐ANCA	S/CO	< 1	6.4	0.4
C3	g/L	0.730–1.460	0.252	0.742
C4	g/L	0.100–0.400	0.016	0.084
IgG	g/L	7.00–17.00	19.06	9.69
RF	IU/mL	0–20	40.3	9.9
EBV VCA IgA	S/CO	≤ 0.80	5.95	0.66
EBV VCA IgM	S/CO	≤ 0.80	1.72	0.30
CMV PP65	cells/2 × 10^5^ leukocytes	Negative	9	4

Abbreviations: ACL, anticardiolipin; ANA, anti‐nuclear antibodies; C3, complement 3; C4, complement 4; CLIA, chemiluminescence immunoassay; CMV, cytomegalovirusEBV, Epstein‐Barr virus; ESR, erythrocyte sedimentation rate; IgG, immunoglobulin G; IIF, indirect immunofluorescence; LF‐ANCA, anti‐lactoferrin antibody; RF, rheumatoid factor; RNP, ribonucleoprotein; rRNP, ribosomal ribonucleoprotein; VCA, viral capsid antigen.

### 2.3 Diagnosis

The patient met the 2019 EULAR/ACR classification criteria for SLE. Based on the criteria, she was assigned 14 points: positive Coombs test (4 points), low C3 and C4 (4 points), and positive anti‐dsDNA antibodies (6 points), exceeding the threshold of ≥ 10 points for classification. System assessment showed involvement of the skin, hematologic system, and joints. Her SLEDAI‐2 K score was 8, derived from arthritis (4 points), low complement (2 points), and positive anti‐dsDNA antibodies (2 points). According to standard definitions (0–4: inactive, 5–9: mild, 10–14: moderate, ≥ 15: severe), this indicated mild disease activity. She was diagnosed with mild active SLE.

### 2.4 Treatment and Clinical Course

The patient was initiated on immunosuppressive therapy with high‐dose prednisolone (60 mg/day) and belimumab. Drug‐induced allergic symptoms occurred during intravenous ganciclovir therapy for CMV antigenemia. Consequently, the antiviral therapy was switched to oral valganciclovir, and cetirizine was added for symptomatic relief. The patient's condition improved steadily, and she was discharged on April 23, 2025. The relative contribution of each agent cannot be determined from a single case. It is acknowledged that high‐dose glucocorticoids and belimumab alone could account for much of the clinical improvement. However, the rapid decline in CMV antigenemia following ganciclovir suggests a possible adjunctive role for antiviral treatment.

### 2.5 Follow‐Up

**FIGURE 1:**
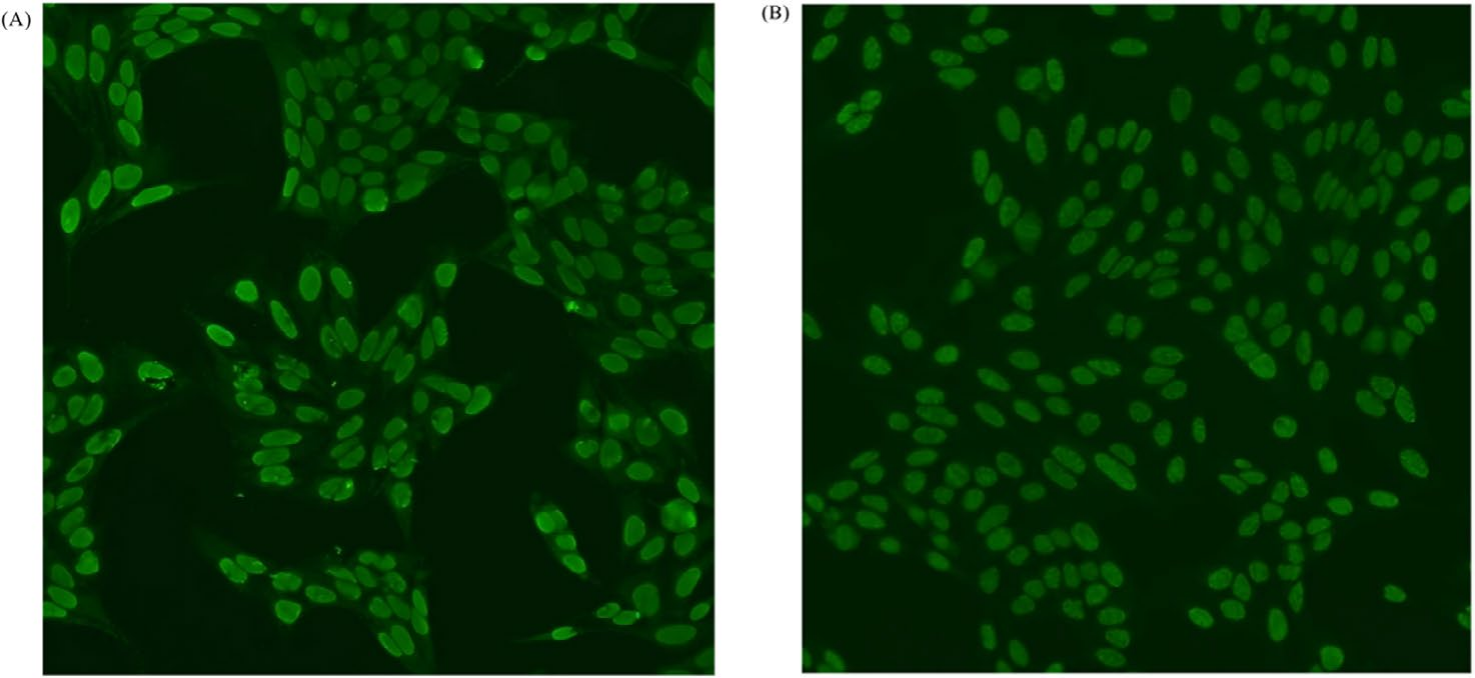
Immunofluorescence patterns observed in a 10‐year‐old SLE patient using indirect immunofluorescence (IIF) on HEp‐2 cells (diluted 1:100) at two time points: (A) April 9; (B) May 28.

At a follow‐up visit on May 28, 2025, the patient showed marked clinical improvement. The facial rash had completely resolved, and she had a normal range of motion in all joints without pain or swelling. Follow‐up laboratory tests demonstrated near‐complete normalisation of inflammatory markers and a significant reduction in autoantibody titers, along with resolution of acute viral markers (detailed in Table 1 and Figure 1).

## 3 Discussion

SLE is a chronic autoimmune disease characterized by multi‐organ damage. Its core pathology involves autoantibodies (e.g., anti‐nuclear antibodies, anti‐dsDNA antibodies) attacking host tissues, immune complexes deposition, and complement consumption [6]. The disease predominantly affects women of childbearing age (15–45 years), accounting for approximately 90% of cases, while pediatric patients constitute 15%–20% and often present with more severe manifestations [7]. The onset of SLE is a complex interplay of genetic predisposition and environmental triggers, with factors like ultraviolet radiation and EBV infection known to induce the disease [8].

This case highlights a compelling co‐pathogenic pattern in a pediatric patient with SLE, evidenced by the simultaneous detection of acute EBV infection (EBV IgM/VCA positive) and CMV antigenemia [9]. A core pathogenic mechanism in this patient is molecular mimicry mediated by EBV. The EBV nuclear antigen EBNA‐1 contains a sequence (PPGGRRP) with high homology to SLE autoantigens (Sm B/B′ and Ro/SSA), which may induce cross‐reactive antibodies that attack host tissues [4]. This is supported by the patient's markedly elevated anti‐dsDNA IgG levels (2684.3 IU/mL) and positive polyclonal autoantibodies (RNP/rRNP). Furthermore, EBV contributes to B lymphocyte dysregulation, as its latent membrane protein LMP1, promoting B cell proliferation and autoantibody production. This is consistent with the patient's elevated B lymphocyte count (15.2%), increased IgG levels, and severe complement depletion (C3 0.252 g/L, C4 0.016 g/L). EBV also amplifies autoimmune responses by disrupting B‐cell tolerance checkpoints and upregulating B‐cell activating factor (BAFF). Finally, CMV appears to synergistically exacerbate the condition by reactivating EBV6 via Toll‐like receptor (TLR) pathways, which release pro‐inflammatory cytokines such as IFN‐α and TNF‐α, creating a vicious inflammatory cycle. The patient's CMV antigenemia, accompanied by fever, arthritis, and low complement levels, suggests viral collaboration in accelerating immune complex deposition and vascular damage.

The remarkable clinical response to belimumab combined with antiviral therapy provides strong support for this pathogenic model. By targeting the BAFF pathway, belimumab inhibited EBV‐driven B‐cell hyperproliferation and indirectly suppressed viral reactivation by reducing the inflammatory burden. Concurrently, ganciclovir effectively controlled CMV antigenemia (reducing it to 4 positive cells/2 × 105 leukocytes), disrupting the inflammatory cascade triggered by the CMV‐TLR pathway and preventing synergistic damage with EBV.

The patient's clinical response to belimumab plus antiviral therapy is consistent with this hypothesis. Given the observational nature of a single case, causality cannot be established. The proposed mechanisms remain hypotheses that warrant further investigation. We searched PubMed and Web of Science up to June 2026 using keywords: (“pediatric” OR “child” OR “adolescent”) AND (“SLE”) AND (“EBV” OR “CMV”). English‐language case reports/series with detailed data were included. This yielded 6 EBV‐associated cases and 4 CMV‐associated cases (Table 2) [10, 11, 12, 13, 14, 15, 16, 17, 18, 19]. Analysis of the six EBV‐related cases from the literature reveals a consistent pattern. Among these six patients, the median age was 11.5 years (range 2–14 years), with females comprising 83.3% (5/6). Common presenting symptoms included fever, arthralgia, pancytopenia, and skin rashes (83.3%, 5/6). Infectious mononucleosis features such as lymphadenopathy and hepatosplenomegaly were observed in 66.7% (4/6) of cases. All diagnoses were confirmed by serological or nucleic acid testing. Notably, antinuclear antibodies (ANA), anti‐dsDNA antibodies, and hypocomplementemia were consistently positive in all cases. Complications included lupus nephritis (50%, 3/6), central nervous system involvement (33.3%, 2/6), and hemophagocytic lymphohistiocytosis (HLH) (33.3%, 2/6). Treatment protocols universally included glucocorticoids, often combined with immunosuppressants (66.7%, 4/6) and biologics (16.7%, 1/6). Symptomatic remission was achieved in 83.3% (5/6) of cases, with no reported fatalities.

**TABLE 2:** Summary of pediatric SLE cases associated with EBV or CMV infection in the literature and the present case.

References	Age/Sex	Symptoms & signs	EBV/CMV infection	Lab findings	Organ involvement	Therapy	Outcome
Brogna et al. (2016)	7y/M	Walking difficulties, fever, pharyngitis, chorea, hallucinations, seizures, hemiplegia	EBV reactivation	Initial neg autoantibodies; later ANA 1:80, dsDNA 22.4 UA/ml, AuNA 120 UA/ml	Nervous system, skin, joints, oral ulcers	IVIG, hormone discontinuation	Remission
Isome et al. (2005)	11y/F	Fatigue, headache, proteinuria, hypertension, severe thrombocytopenia, hemolytic anemia	EBV reactivation	ANA 1:1280, dsDNA 160 U/mL, low C3/C4, hemophagocytes in BM	Kidney (LN), hematologic	IVIG, antibiotics, protease inhibitors	Remission
Petrea et al. (2024)	14y/F	Fatigue, headache, hair loss, amenorrhea, rash, weight loss (20.4%), fever, oral ulcers	EBV + SARS‐CoV‐2	ANA > 1750 AU/mL, dsDNA > 400 IU/mL, low C3 (40 mg/dL), low C4 (< 8 mg/dL)	Blood, kidney (Stage II), skin, digestive	Prednisolone, prednisone, belimumab, azathioprine	Improvement
Kasapcopur et al. (2006)	14y/F	Generalized rash, fever, headache, eyelid edema, lymphadenopathy, hepatosplenomegaly, ecchymosis	EBV infection	ANA+, dsDNA 6.5 AU/mL, low C3/C4, proteinuria 1.16 g/24 h	Blood, kidney (LN IIB), skin	Glucocorticoids, cyclophosphamide	Remission
Cheawcharnpraparn et al. (2024)	12y/F	Fever, epilepsy, altered consciousness, hallucinations, hepatosplenomegaly, oral ulcers, proteinuria	EBV cerebritis	ANA 1:1280, dsDNA+, low C3/C4	CNS (encephalitis), hematologic (HLH)	IVIG, cyclosporine A, prednisone	Gradual improvement
Kishi et al. (2013)	2y/F	Fever, malar rash, arthritis, proteinuria	EBV primary infection	ANA+, anti‐dsDNA+, low C3/C4	Kidney (LN), skin, joints	Glucocorticoids, cyclophosphamide	Remission
Lan et al. (2024)	2 m/M	Oral candidiasis, facial erythema, persistent liver impairment	CMV infection	ANA 1:3200, SSA+, dsDNA+, elevated ALT/AST/TBA	Liver, skin	Hepatoprotective agents	Remission, autoantibodies negative
Barros et al. (2021)	9y/F	Shortness of breath, joint pain, fever, rash, petechiae, cardiac tamponade	CMV infection	ANA+, dsDNA+, low C4, +direct Coombs	Heart (pericardial tamponade), joints, skin, blood	Ganciclovir, prednisone, azathioprine, IVIG	Improvement
Yamazaki et al. (2015)	12y/F	Recurrent fever, cervical lymphadenitis, abnormal liver function, butterfly rash, palmar erythema	CMV infection	ANA 1:640, dsDNA 119 IU/mL, low C3/C4, proteinuria 2.4 g/24 h	Kidney (LN III), liver, skin	Ganciclovir, prednisone, methylprednisolone, cyclophosphamide	Good condition
Sankar et al. (2008)	15y/M	Repeated fever, fatigue, epilepsy, hepatosplenomegaly, otitis media, persistent lymphocytosis (95%)	CMV infection	ANA 1:160, dsDNA 54 IU/mL, predominant lymphocytosis	Blood, nervous system	Ganciclovir	Remission
Our case (present)	10y/F	Butterfly rash, joint swelling/pain, fever	Acute EBV + CMV co‐infection	ANA 1:1280, dsDNA 2684.3 IU/ml, low C3/C4, elevated IgG, +Coombs	Skin, joints, blood	Prednisolone, belimumab, ganciclovir	Remission

Similarly, an analysis of the four reported CMV‐associated pediatric SLE cases and this clinical report shows common features. Among the five children with CMV‐associated SLE, the median age ranged from 2 months to 15 years, with a male‐to‐female ratio of 2:3. Most cases exhibited fever (80%), along with skin rashes, liver dysfunction, and cytopenia (60%). Cardiac involvement was also reported (20%). All diagnoses were confirmed by serological, nucleic acid, or antigenemia assays (CMV PP65). ANA and anti‐dsDNA antibodies were positive in 100% and 80% of cases, respectively, with concurrent hypocomplementemia. Complications included lupus nephritis (40%), central nervous system involvement (20%), and myocardial damage (20%). All patients received antiviral agents (e.g., ganciclovir), with most also receiving glucocorticoids (80%) and immunosuppressants (40%). Symptom remission was achieved in 80% of cases, with no fatalities reported (detailed in Table 2).

## 4 Conclusion

This case report suggests a possible contribution of acute EBV and CMV infections to SLE pathogenesis through distinct but potentially synergistic mechanisms, supporting the “viral infection‐autoimmunity” hypothesis. However, these interpretations remain hypotheses, and causality cannot be established from a single case report. Limitations include single‐case design, absence of quantitative PCR confirmation for EBV and CMV, lack of long‐term follow‐up, and retrospective analysis.

## Author Contributions

Anning Chen, Gengchun Ji and Shulan Zhang conceived and designed the study. Ziping Lan, Jianwei Wang and Le Zhang collected and compiled the clinical data. Anning Chen and Gengchun Ji contributed to literature review and diagnostic interpretation. Anning Chen and Gengchun Ji drafted the manuscript. All authors critically revised the manuscript, approved the final version, and agree to be accountable for all aspects of the work.

## Funding

This work was supported by Peking Union Medical College Hospital Talent Cultivation Program (Category C, no. UBJ06102).

## Ethics Statement

The study protocol received approval from the Ethics Review Committee of Peking Union Medical College Hospital (Project no. K4435).

## Conflicts of Interest

The authors declare no conflicts of interest.

## Data Availability

The datasets supporting the findings of this case report are contained within the article. The original medical records are not publicly available due to patient privacy concerns but can be accessed from the hospital by a formal request and with ethics approval.
